# Diagnosis of Langerhans Cell Histiocytosis on Fine Needle Aspiration Cytology: A Case Report and Review of the Cytology Literature

**DOI:** 10.4061/2011/439518

**Published:** 2011-01-20

**Authors:** Neeta Kumar, Shahin Sayed, Sudhir Vinayak

**Affiliations:** ^1^Department of Pathology, Aga Khan University Hospital, Third Parklands Avenue, P.O. Box 30270, GPO 00100, Nairobi, Kenya; ^2^Department of Radiology, Aga Khan University Hospital, Third Parklands Avenue, P.O. Box 30270, GPO 00100, Nairobi, Kenya

## Abstract

A case of multifocal Langerhans cell histiocytosis in a two-year-old child is presented where fine needle aspiration was helpful in achieving a rapid and accurate diagnosis in an appropriate clinical and radiological setting. This can avoid unnecessary biopsy and guide the management especially where access to histopathology is limited. The highly characteristic common and rare cytological features are highlighted with focus on differential diagnoses and causes of pitfalls.

## 1. Introduction

Langerhans cell histiocytosis (LCH) is a rare disease affecting predominantly children. It can present as a solitary lesion requiring no treatment or as a multisystem, life-threatening disorder necessitating aggressive therapy [1]. 

We present a case of LCH in a child where fine needle aspiration (FNA) was helpful in establishing a rapid and correct diagnosis in correlation with radiology. The purpose is to highlight common and rare cytological features. This will add to the pathologist's confidence in rendering a rapid and accurate cytologic diagnosis, avoid unnecessary biopsy and guide appropriate management. This is especially valuable in a setting where cytopathologist expertise may not be easily available and histopathology services are located only in big cities and are inaccessible to patients in rural areas due to the long distance and high cost involved.

## 2. Case Report

A two-year-old female child presented to the outreach centre of our university hospital with swellings on right frontal and occipital regions of skull for the last one year. On examination, these were fluctuant, ill-defined soft tissue masses which measured 2 × 2 and 3 × 3 cms, respectively. In addition, a cervical lymph node was palpable on the left side measuring 1 × 1 cms. It was firm, tender, and slightly mobile. The patient had no fever or loss of weight. The liver and spleen were not palpable. 

Peripheral blood film showed microcytic hypochromic anemia. Hemoglobin was 10 gm/dL. Differential count showed 19% monocytosis with 38.3% neutrophils, 38.9% lymphocytes, 2.5% eosinophils, and 1.3% basophils. Platelet count was normal. The initial clinical impression favored a malignant lesion. The patient was referred for FNA. 

FNA from lymph node yielded whitish aspirate. FNA from right frontal and occipital masses yielded 0.5 mL and 1 mL hemorrhagic fluid, respectively. The fluid was centrifuged to make smears from the sediment. Ethanol-fixed smears and air-dried smears were prepared and stained with Papanicolaou and Giemsa method, respectively. The remaining sediment was processed to make cell block for immunochemistry. 

**Figure 1:**
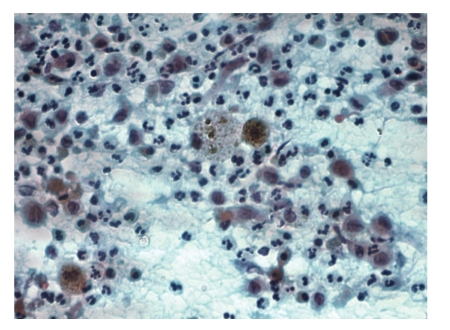
FNA smear from frontal mass showing single and loosely clustered Langerhans cells admixed with neutrophils, lymphocytes and, reactive histiocytes. Two foamy macrophages containing hemosiderin are seen in the centre (Papanicolaou stain, original magnification, ×400).

Smears were highly cellular and showed numerous atypical histiocytes as the predominant cell type scattered singly and in loosely cohesive clusters. These were admixed with a polymorphic population of eosinophils, neutrophils, lymphocytes, plasma cells, foamy histiocytes, and multinucleated reactive histiocytic giant cells (Figure 1). Smears from both the swellings in the skull and cervical lymph node were morphologically similar except that atypical histiocytes were less in number and eosinophils were more abundant in smears from lymph node as compared to smears from skull lesions.

**Figure 2:**
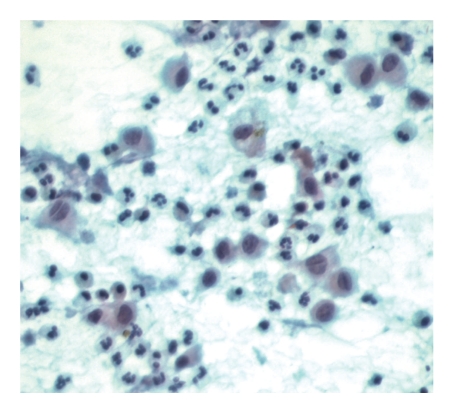
Langerhans cells with moderate to abundant cytoplasm and prominent nuclear grooves (Papanicolaou stain, original magnification, ×400).

The atypical histiocytes were large cells with moderate to abundant, pale blue cytoplasm and an eccentric or central round to oval, vesicular nuclei. Prominent nuclear indentations and grooves (with a coffee bean appearance) were observed which were best seen in Papanicolaou stain (Figure 2). Some showed intranuclear pseudoinclusions. Nucleoli were absent. These cells displayed marked pleomorphism with variation in size and shape of cells and nuclei. Occasional mitoses were seen. Some of these cells showed cytoplasmic processes. Most were mononuclear, and some were binucleate or multinucleated. The multinucleated giant cells had complex folded nuclei similar to mononuclear atypical histiocytes and were easily differentiated from reactive multinucleated histiocytic giant cells.

**Figure 3:**
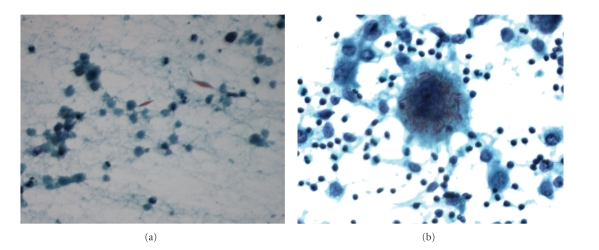
(a) Extracellular rhomboid Charcot-Leyden crystals (Papanicolaou stain, original magnification, x400). (b) Macrophages with several ingested Charcot-Leyden crystals (Papanicolaou stain, original magnification, x400).

The multinucleated reactive histiocytic giant cells contained numerous indented vesicular nuclei in abundant cytoplasm. They also contained hemosiderin in smears from skull masses (Figure 1). In addition, many rhomboid and needle-shaped Charcot-Leyden crystals were seen both extracellular (Figure 3(a)) and intracellular in the giant cells (Figure 3(b)). The atypical histiocytes stained positive for both cytoplasmic and nuclear S-100 protein. The cytologic findings were highly suggestive of LCH.

At this point, a plain X-ray was requested which showed two lytic lesions corresponding to occipital and frontal swellings. Subsequently, computerized tomogram (CT) with 3D reconstruction showed lytic lesions which were clearly demarcated “punched-out” lesions (the classic geographic skull) in frontal and occipital regions. There was associated homogenous soft tissue swelling of the scalp but no breech of the dura. No other systemic involvement was found.

## 3. Discussion

LCH is a rare disease, and the estimated annual incidence ranges from 0.5 to 5.4 cases per million persons [2]. In the past, the disorder was referred to as histiocytosis X and had three variants: eosinophilic granuloma, Hand-Schuller-Christian disease and Letterer-Siwe syndrome. These three conditions are believed to represent different expressions of the same disorder, now known as LCH [3, 4]. 

An ongoing debate exists over whether this is a reactive or neoplastic process [2]. The disease is characterized by a clonal proliferation of the antigen-presenting dendritic cell called the Langerhans cell (LC) [5, 6]. The proliferation may be induced by a viral infection, a defect in T cell-macrophage interaction, and/or a cytokine-driven process mediated by tumor necrosis factor, interleukin 11, and leukemia inhibitory factor [2, 7–10]. 

LCH may occur at any age, although the majority of the cases are diagnosed in children from newborn to 15 years. There is no significant gender difference. The clinical spectrum varies from a solitary lesion, to multifocal unisystem to multisystem lesions with related symptoms. The unifocal form usually involves the bone, often seen in children between 5 and 15 years old. Systemic LCH is more common in children under 2 years of age. The multifocal unisystem form almost always occurs in the bone. Any bone can be involved, but more than 50% of lesions occur in the skull, spine, pelvis, ribs, and mandible. The multifocal multisystem form involves many organs, including the bone, skin, liver, spleen, hematopoietic system, and lymph node [2, 11]. The lymph node involvement in LCH can be seen as a part of a systemic disease or as a localized lesion, although isolated nodal involvement is rare. Lymph node may also enlarge as a reaction to bone or skin lesions [12]. 

Traditionally, the diagnosis of LCH is based on hematologic and histologic criteria [2, 4, 13–15]. Enough experience has accumulated in accurate cytological diagnosis of LCH in various body sites on the basis of characteristic cytological features in the presence of appropriate clinical and radiological setting as evident from several case reports and case series [16–31]. Study of these shows that cytology closely reflects histomorphology. Ancillary studies may not be always necessary for diagnosis in appropriate setting [32]. 

The classical cytological features include high cellularity composed of sheets and many isolated LCs seen admixed with polymorphous population of numerous eosinophils, neutrophils, lymphocytes, plasma cells, multinucleated giant cells, and macrophages. The key to the diagnosis is to identify the LC through its characteristic features, namely, nuclear grooves and nuclear pseudoinclusions. They show variable degree of pleomorphism and mitotic activity [17, 18, 22, 26, 29]. Presence of dendrite-like cytoplasmic processes in LCs is a rare but characteristic feature [22, 33, 34]. Sometimes the LCs are few or nuclear grooves not very prominent or lack cytopalsmic processes. Degree of eosinophil infiltration varies in different areas of LCH lesion and different organs, thus their number can vary from scant to abundant in cytology smears [22]. Their presence can help attract attention to the diagnosis. In our case, eosinophils were more abundant in lymph node smears as compared to skull lesions which had more of LCs and reactive histiocytes. 

Presence of Charcot-Leyden crystals singly and in bunches within the macropahges, giant cells, and extarcellularly was a unique feature in our case and has been reported very rarely [20, 27, 29, 31]. Charcot-Leyden crystals are crystalloids containing eosinophil membrane protein formed from rupture of eosinophil's granules. They indicate tissue eosinophilia and may help in drawing attention to the LCH diagnosis. 

The diagnosis of LCH in our patient was made on the basis of FNA which showed characteristic (both common and rare) features of LCH. This was corroborated by characteristic radiology and clinical findings. In this case, CT showed lytic lesions in the skull bones having sharp borders with a punched-out appearance. Destruction of both the inner and outer tables results in a double-contour or beveled-edge appearance which is a typical feature in the diagnosis of LCH [35, 36]. 

The cytologic diagnosis may be missed due to lack of familiarity with its cytological features among pathologists or due to the lack of characteristic cytological findings resulting from a sampling error. Therefore it is prudent on the part of the pathologist to consider this diagnosis only in an appropriate clinical and radiological setting. It is also necessary to be familiar with cytological features of other differential diagnoses.

In the present case, the most common differential diagnoses of skull lesions clinically included Ewing's sarcoma, non-Hodgkin lymphoma, and osteomyelitis. Ewing's sarcoma and non-Hodgkin lymphoma are characterized by monotonous population of small round blue cells. In acute osteomyelitis, the neutrophils form a prominent component. The reactive histiocytes are seen and can be easily distinguished due to the absence of distinctive features of LCs. Chronic osteomyeltis shows predominantly plasma cells and lymphocytes. Plasma cells and neutrophils are infrequent in LCH.

Sinus histiocytosis with massive lymphadenopathy (SHML) involves primarily the cervical nodes, but its histiocytes are morphologically quite different from those of LCH. In SHML, the histiocytes have abundant cytoplasm, exhibiting hematopoietic phagocytosis and prominent nucleoli [28].

Secondary hyperplasia of the LCs is associated with lymphomas, especially with Hodgkin's disease and lung tumors. Care should be taken to differentiate these hyperplastic Langerhans cells from atypical LCs of LCH. Rarely, LCH can be associated with another malignancy such as malignant lymphoma, leukemia, or metastatic neoplasm [37, 38]. These need to be excluded after a diligent search for malignant cells with obvious cytologic atypia in the smear. Malignancies with tumor cells commonly having nuclear grooves or pseudoinclusions should also be considered, such as malignant melanoma and papillary thyroid carcinoma.

LCs show positivity for S-100, PNA (peanut agglutinin), MHC class II, CD1a, and langerin (CD207) [2]. Our case showed positivity for S-100 protein. CD1a and langerin are not available in our lab. The Birbeck granule is their distinctive ultrastructural hallmark [2]. Electron microscopy was not performed in our patient and was not considered essential for diagnosis as also suggested by other authors [32]. 

Patients with apparently restricted LCH need careful staging of their disease to ensure that the lesions are not part of a more extensive process. FNA can be used to establish the extent of disease or recurrence of LCH [18]. In children with multiple swellings as in our case, FNA, being minimally invasive, is particularly suitable to sample all swellings in detecting the extent of involvement. For localized lesions in the skeletally immature patients, a simple, minimally invasive form of treatment with a low rate of complication is desirable. In view of this and the possibility of spontaneous resolution in localized disease, FNA alone could be used to confirm the diagnosis. 

To conclude, the present case highlights the role of FNA in the diagnosis of the rare disease of LCH in a child with usual clinical presentation. The cytologic features of LCH are highly characteristic to suggest a diagnosis in an appropriate clinical setting with classical radiological findings. A high index of suspicion, awareness of common and rare cytological features of LCH, its differential diagnoses, and causes of diagnostic pitfalls is necessary. This can obviate the need of biopsy and electron microscopy. Immunochemistry if available can be performed on cell block.
